# Minimally invasive balloon-assisted sinus floor elevation vs. conventional transcrestal procedure in terms of new bone formation in a split-mouth Goettingen minipig model

**DOI:** 10.1186/s40729-024-00546-x

**Published:** 2024-12-18

**Authors:** Andreas Kolk, Florian Bauer, Jochen Weitz, Robert Stigler, Benjamin Walch, Florian Grill, Marko Boskov

**Affiliations:** 1 Department of Oral and Maxillofacial Surgery, Klinikum Rechts der Isar, Technical University of Munich, Munich, Germany; 2 Department of Oral and Maxillofacial Surgery, Ludwig-Maximilians University, Munich, Germany; 3 Department of Oral and Maxillofacial Surgery, Med University of Innsbruck, 6020 Innsbruck, Austria

**Keywords:** Open sinus floor elevation, Transcrestal balloon technique, Implant stability, Easy-graft, Bone formation

## Abstract

**Purpose:**

Currently, maxillary sinus floor (SF) elevation is based on off-the-shelf allogeneic, xenogeneic or synthetic bone augmentation materials (BAM) that are implanted via an open lateral sinus wall approach (OSFE). However, this invasive method is associated with postoperative complications caused by an inadequate blood supply of the alveolar ridge. Balloon-assisted procedures are minimal invasive alternatives with lower complication rates. The aim was to evaluate local new bone (NB) formation in the SF following the application of a particulate BAM (Easy graft) via two different SF elevation techniques in a split mouth mini-pig sinus augmentation model.

**Material and methods:**

Seven adult Goettingen minipigs were used for evaluation of a biphasic ceramic (PLGA/ß-TCP) BAM in the elevated SF region. Treatments were randomized to the contralateral sinus sites and included two procedures: OSFE (control group) versus minimally invasive SF elevation by a balloon-lift-control system (BLC) (treatment group). The animals were euthanized after 28 and 56 days for analysis of new bone (NB) formation.

**Results:**

The biphasic synthetic BAM implanted via BLC increased more NB formation (5.2 ± 1.9 mm and 4.9 ± 1.6 mm vs. 2.6 ± 0.5 mm) and osseointegration of the particles (18.0 ± 6.0% and 25.1 ± 18.2% vs. 10.1 ± 8.0%, p < 0.05) compared to the control.

**Conclusions:**

Implantation of a biphasic synthetic BAM enhanced NB formation in the mini-pig maxillary sinus at both time points and in both groups, although BLC resulted in a slightly better total NB formation compared to the control.

**Graphical abstract:**

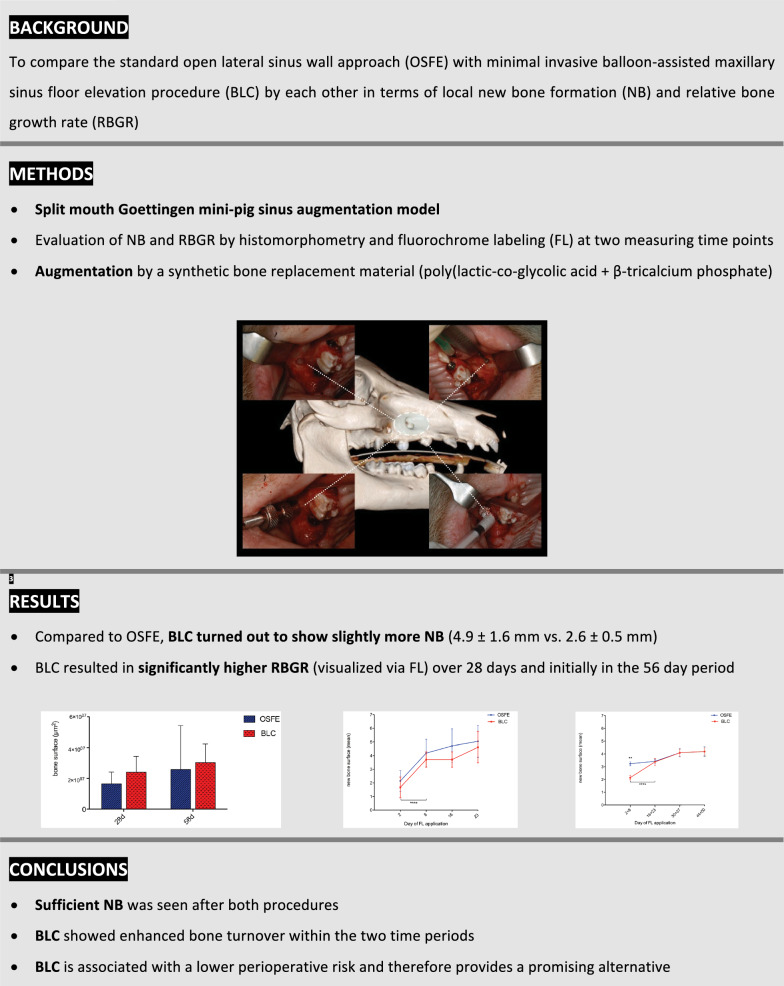

## Background

Sinus floor (SF) elevation for bone generation is a common surgical procedure prior to dental implantology, leading to increased quality of life [28]. Therefore, not only enhanced bone augmentation materials (BAM) but also different techniques are propagated [27].

The conventional surgical approach represents external osteotome SF elevation (OSFE) according to Tatum: surgical access is made via a vestibular bony window in the lateral part of the sinus [32]. Therefore, the implant-bearing alveolar crest remains undamaged. By preparing the lateral bony window, the Schneiderian membrane can be lifted, and BAM is applied into the newly formed cavity. However, this technique requires advanced surgical skills to maintain mucosal integrity [6, 14] and is often accompanied by the disadvantage of eventual resorption processes caused by inadequate postoperative blood supply attributable to wide deperiostation [2, 8, 20, 30, 32].

When the balloon‐assisted sinus lift (SL) procedure (Balloon-Lift-Control- (BLC-) System) was initially proposed in 2006 it became popular mostly due to the minimal membrane perforation rate and successful and technically easy application of the balloon even in anatomically complex regions. This approach is expected to show fewer postoperative complications, such as bleeding, discomfort, swelling and infections [4].

The final evaluation of the latter technique especially in terms of the amount of bone augmentation is still required [2].

Therefore, in this study, the abovementioned SF technique performed by the BLC-system (treatment-group, TG) was compared with classic open SF elevation procedure (control-group) in a split mouth animal model. Here in the TG the SF is burred carefully at the implantation site before a greenstick fracture of the mucosal part of the compacta is induced. Thus, the SF can be carefully elevated by a balloon catheter. The BAM is then applied into the gap to obtain [30]. This minimally invasive technique appears to be advantageous in many ways. The mucosa is elevated in a plane fashion with equal pressure distribution, by which it remains undamaged, and the risk of inadequate blood perfusion due to vascular stress of the Schneiderian membrane is reduced [25]. In addition, due to the lack of interference with underwood septa a significant lower risk of severe membrane perforations occurs [33]. Apart from the surgical technique, the biology of the BAM and other clinical factors, such as chewing forces, patient compliance, smoking behavior, and comorbidities, also play an important role in successful bone regeneration.

[17].

To reduce potential biases, SF elevation was performed in a split mouth model on Goettingen minipigs bilaterally under equal terms (same surgeon, same amount of BAM). To better determine the effect of the invasiveness of sinus access and the membrane lifting procedure, this histological study was performed.

## Materials and methods

### Material

BAM Easy graft is an osteoconductive microporous synthetic material with varying pore diameters from 1–10 μm and consists of a copolymer coating with poly(lactic-co-glycolic acid) (PLGA) and the BAM β-tricalcium phosphate (β-TCP). In addition, a biolinker is activated by blood contact during the first hours after application and is subsequently removed (90%) from the bone substitute material within three hours. Subsequently by hydrolytic cleavage of the polymer chains the PLGA coating is biodegraded over a few weeks’ parallel to the healing and bone ingrowth process gradually replacing the β-TCP.

### Animals

**Fig. 1:**
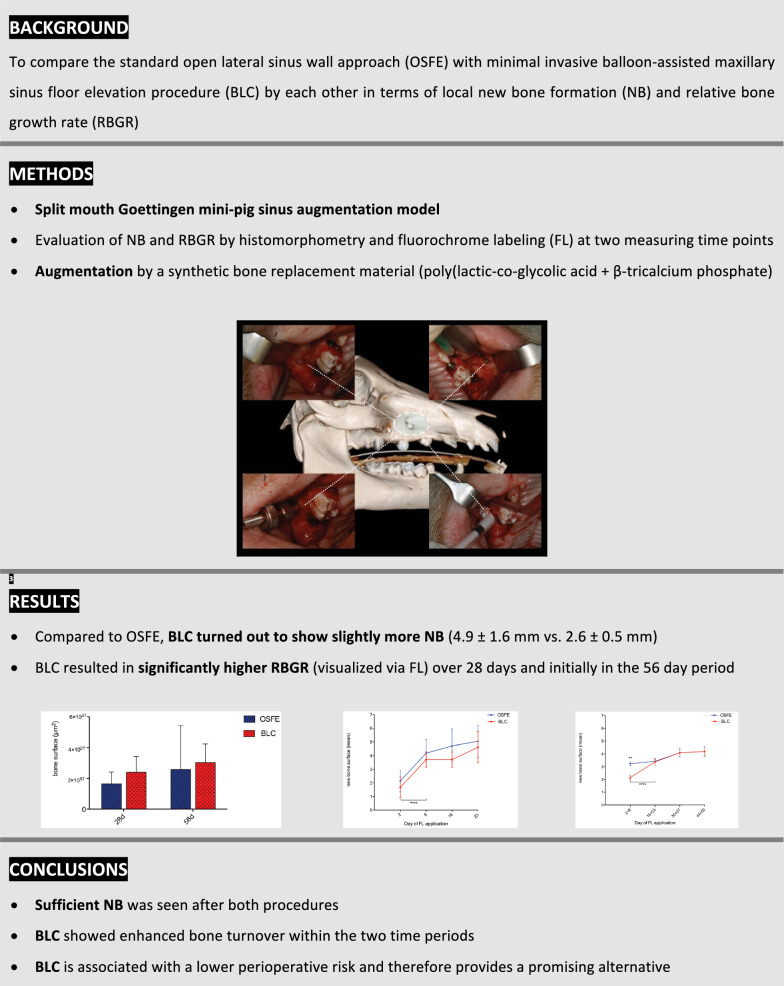
(Results, left graph) Histomorphometric measurements of the new bone surface in both groups: open sinus floor elevation (OSFE) and transcrestal sinus floor elevation with a balloon-lift control system (BLC). Differences not significant. (Results, central graph) Relative bone growth rate (RGBR) by FL over 28 days: Growth rate is significantly higher between day 2 and day 8 for the BLC group: ^****^p = 0.0001. The growth rate decreases for the BLC group after 8 days and then increases after 16 days with a wide standard deviation. (Results, right graph) RGBR by FL over 56 days: Growth rate is significantly higher between the first two fluorescent dyes applied for the BLC group: ^****^p = 0.0001. After 23 days, the growth rate decreases for both groups; there is almost no change measurable after 37 days in both groups. A significant difference in NB growth rate between the two therapy groups could be measured at the initial timepoint: ^*^p = 0.03

**Fig. 2:**
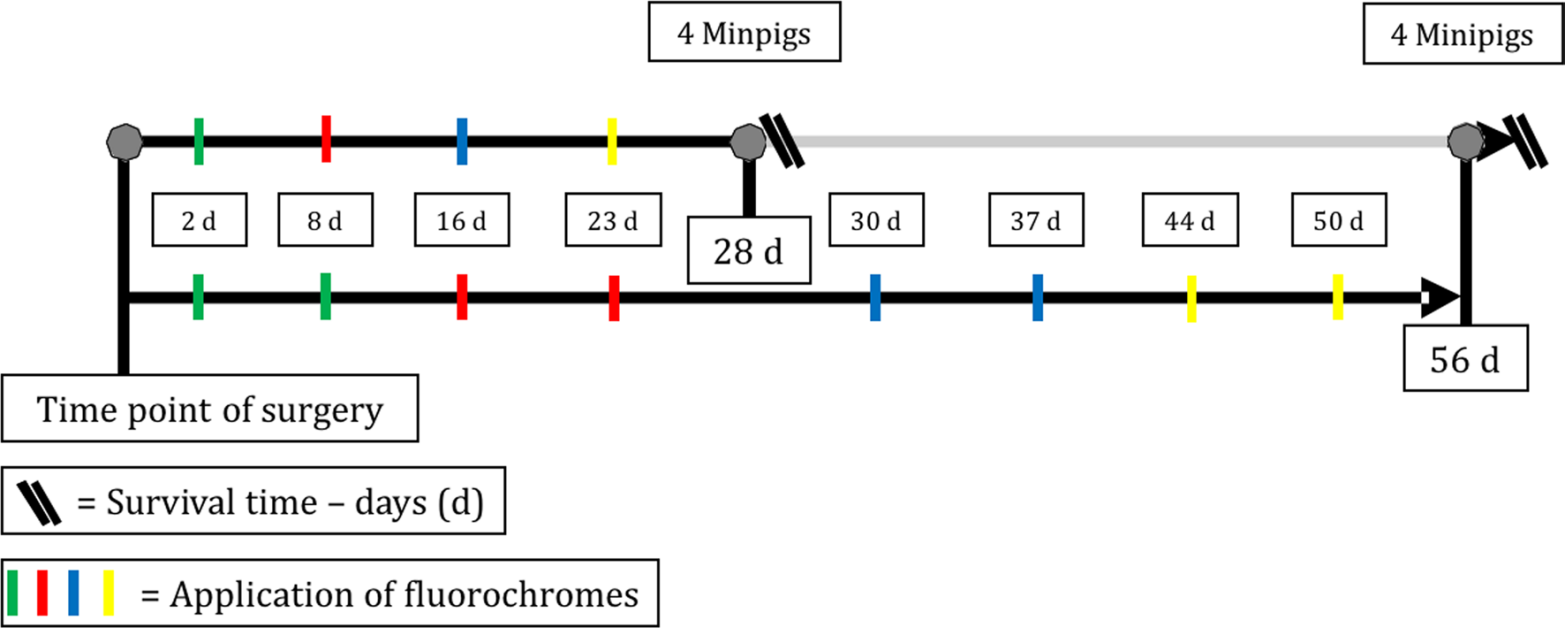
Timeline of the fluorescent dye application at the different time points: Calcein green (Cg, green), Xylenol orange (X, red), Calcein blue (Cb, blue) and Rolitracycline (R, yellow). The animals in the 56-day group received a double dose of each fluorescent dye

To investigate the effect of SF augmentation in terms of the local distribution of new bone (NB) formation, seven Goettingen mini-pigs were operated upon in a split mouth setting (Fig. 1). One randomly chosen sinus cavity per animal was operated upon with the OSFE technique (control group), whereas the BLC technique was applied to the contralateral sinus cavity (treatment group). For time-dependent analysis, three animals were sacrificed 28 days after surgery, and the remaining 4 animals 56 days after surgery. To evaluate NB and RBGR, animals received in vivo fluorescence labeling (for details, see Fig. 2) for further histomorphometric analysis and fluorescence microscopy.

### Animals and anesthesia

The study was performed on 7 Goettingen minipigs obtained from Ellegaard Gottingen Minipigs (Dalmose, Denmark) weighing an average of 22.5 (range 19.0–28.2) kg and having a mean age of 14.7 (range 13.0–16.5) months.

After approval by the local animal legislative committee (reference number: 55.2-1-54-2531-119-07), all experiments were performed according to the local and legal guidelines. Housing and animal care were executed at the Centre for Preclinical Research at the Technical University of Munich in accordance with EU Directive 86/609. In addition, the animal study complied with the ARRIVE guidelines and the European Union Directive 2010/63/EU for animal experiments.

The animals were operated on under general anesthesia. For interventional procedures, a controllable reflex-free general anesthetic with artificial respiration in a semi-closed circulatory system was applied. Premedication was performed with 2 mg/kg azaperone, 10–15 mg/kg ketamine and 0.02 mg/kg atropine.

After sedation, general anesthesia was initiated with 1% propofol (3–5 mg/kg) until intubation capability. After intubation, the animal was ventilated artificially. The anesthetic was maintained with 2% propofol as a continuous drip infusion (~ 7 mg/kg/h). Prior to all surgical procedures, fentanyl was administered intravenously according to effect. During the entire procedure, the animals were monitored by capnometry, pulse oximetry, ECG and temperature probe. If necessary, ventilation and depth of anesthesia were readjusted.

Carprofen (4 mg/kg) and metamizole (40 mg/kg) were administered intraoperatively to achieve continuous analgesic coverage throughout the procedure and immediately after surgery. Intraoperatively, animals were infused with Ringer's solution (10 ml/kg).

After surgery, the animals were given analgesics (4 mg/kg carprofen) orally for a period of 3 days. The mini-pigs were sacrificed under general anesthesia with pentobarbital (100 mg/kg i.v.) by administration of potassium chloride (> 2 mmol/kg), leading to cardiac arrest.

### Surgical procedure

After randomization, both different SL techniques were performed, as already described by Stelzle and Benner, in the same animal [30].

The OSFE was performed as follows: After submucosal infiltration of 4 ml Articain with Epinephrine, a lateral triangular mucoperiostal flap was raised. With a diamond burr (Meisinger, Germany), a lateral osteotomy was performed at exactly 0.3 × 0.5 cm. After careful dissection of the Schneiderian membrane using blunt SL instruments (Meisinger, Surgical Kit 1), the cavity was filled with 2 ml Easy Graft (60% HA + 40% β-TCP + Biolinker) (Fig. 1).

The BLC consists of a plastic syringe connected with a catheter hold balloon at its end, which is filled with saline solution for testing before being inserted into the created crestal approach. Then, the balloon is gently placed beneath the exposed sinus membrane and slowly inflated, hoping to gradually and uniformly displace the sinus membrane in all directions until achieving an approximate 6 mm height gain. The balloon system was then emptied, and the catheter was withdrawn, following which the BAM Easy Graft was introduced and compacted into the space created.

### Polychrome sequential fluorescent labeling (FL)

The fluorescent markers were applied by the subcutaneous (s.c.) injections of 3% solutions in 2% NaHCl_3_ (pH 7.2, 0.5 ml) in the abovementioned order over a period of 4 and 8 weeks postsurgery under i.m. anesthesia with 250 mg zolazepam and 250 mg tiletamine (Tilest 500, Parke-Davis, Freiburg, Germany).

The 8-week group was double-marked. This means that the fluorescent markers were administered repeatedly at 7 days after the first application. The markers Calcein green (Cg; Sigma‒Aldrich, St. Louis, MO, USA) (green), Xylenol orange (X; Sigma‒Aldrich, St. Louis, MO, USA) (red), Calcein blue (Cb; Sigma‒Aldrich, St. Louis, MO, USA) (blue) and Rolitracycline (R; Sigma‒Aldrich, St. Louis, MO, USA) (yellow) were used. The application timetable is depicted in Fig. 2. Further details are published previously [16, 18].

### Sample preparation

After scarification, maxillary sinuses were carefully dissected and perfused in 4% paraformaldehyde. Next, specimens were dehydrated in a graded series of ethanol (from 70 to 100% [v/v]) and acetone and consecutively embedded in methyl methacrylate (MMA, Technovit® 9100 NEW, Kulzer).

For correct alignment of the cutting planes, CT images of the embedded specimens were taken (Philips Brilliance ICT scanner 256; Philips Deutschland GmbH, 20099 Hamburg; with “inner-ear protocol”; layer thickness 670.00 microns; angle gantry: 0°). To ensure correct transfer of CT data perfect orientation of the MMA blocks, each block was marked with CT dense dental fillings.

### Cutting technique

The 3D-CT data were aligned with the MMA blocks. By a comparison of the CT images and the previously attached markings on the blocks, the cutting angle could be determined precisely. According to the previously defined planes (perpendicular to the surface), trimmed blocks were glued onto a brass holder by using cyanoacrylate glue (Cyanolit 201, Bürklin OHG, D-82041 Oberhaching) and sawed in the desired cutting plane (inner hole saw with a diamond saw blade, Leica 1600SP, D-35578 Wetzlar). Each sample delivered approximately 20 sections.

**Fig. 3:**
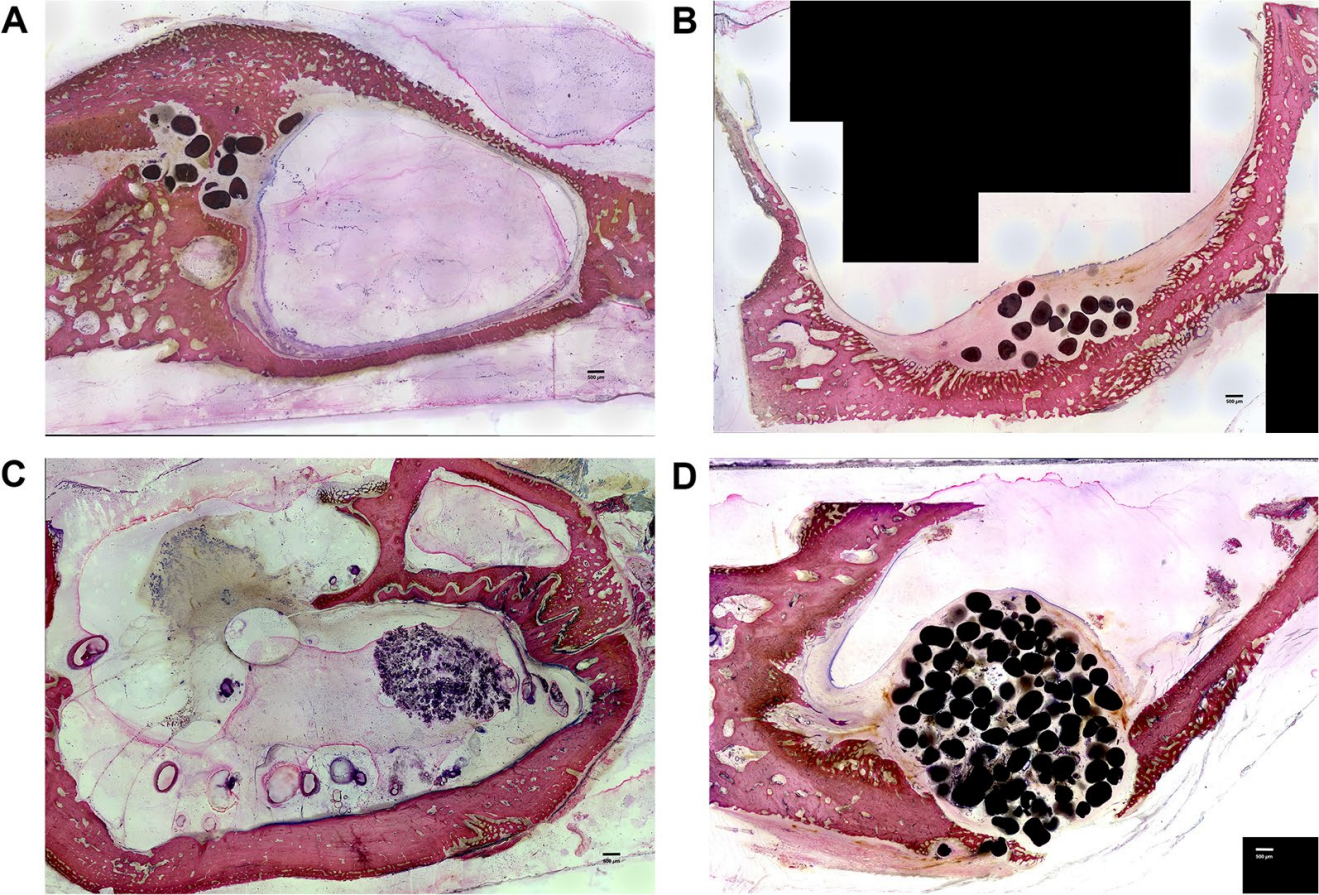
Giemsa eosin staining, samples cut in the coronal direction. **A** Sample of the BLC group at 28 days. The mucosa remained completely intact, and BAM could be positioned gently. Scale bar: 500 µm. **B** Sample of the BLC group at 56 days. The transition zone from local bone to NB can be distinguished by the dark colored NB. Regular osteoid structures are being formed around the BAM particles underneath the mucosa. Scale bar: 500 µm. **C** Sample of the OSFE group at 28 days. The mucosa is detached over the entire sample. NB can be seen at the right superior part of the specimen; however, it is very irregular and not well connected to the original bone. Scale bar: 500 µm. **D** Sample of the OSFE group at 56 days. Mucosal detachment is visible in the right part of the specimen. Scale bar: 500 µm

Approximately 3–4 sections per sample were selected, glued with cyanoacrylate onto a Plexiglas slide, sanded flat by using the Donath wet-grinding process and polished (Leica EM RES102) until a thickness of 80 µm. Of these slides, 1 unstained slide per sample was subjected to fluorescence analysis, and 2 slides each were further proceeded to histological staining. This resulted in a total of 42 sections, 28 of which were evaluated by light microscopy and 14 by fluorescence microscopy. Prior to analysis, a complete microscopic photo was taken of each section, composed of several single images (Fig. 3A–D).

### Histological staining

**Fig. 4:**
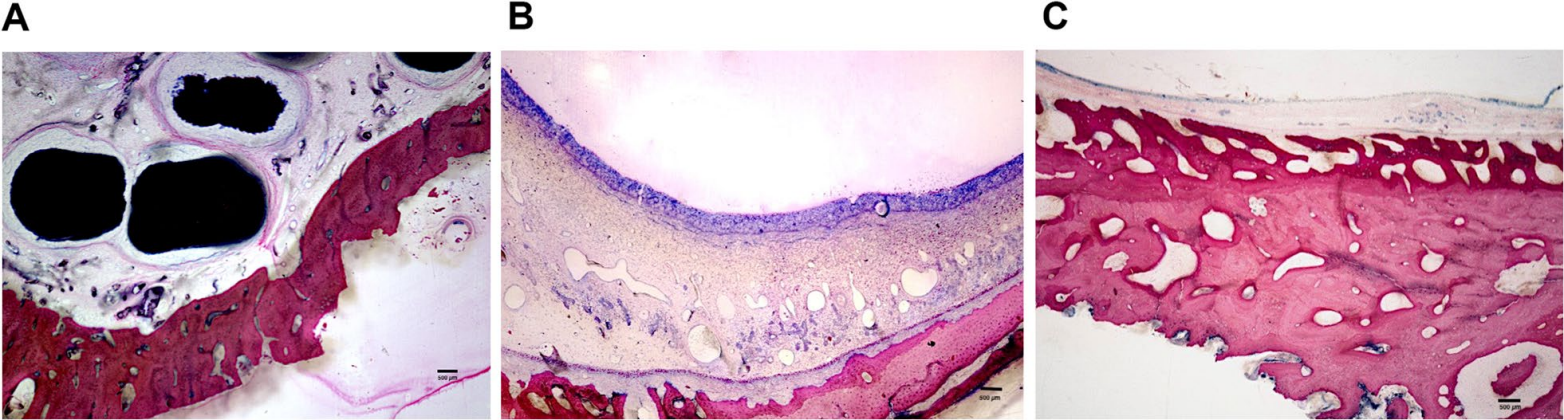
Giemsa eosin staining, coronal detail view. **A–C** Specimen of the BLC group, proving mucosa remaining attached along the entire SF surface, allowing steady and regular regeneration and NB formation (dark red intensely stained regions). Scale bar: 500 µm

Giemsa-eosin staining was chosen for the histological examination of the MMA sections (Figs. 3A–D, 4A–C). The sections were initially etched in 0.1% formic acid for 15 min. Subsequently, Giemsa stain solution (Sigma Aldrich, St. Louis, MO, United States) was applied, after which the specimens were stored in a moist chamber at 60 °C for 30 min. Following a short immersion in 0.1% acetic acid, all sections were thoroughly rinsed, stained for 4 min with 0.1% eosin solution and a drop of glacial acetic acid and then rinsed with distilled water. The eosin solution was diluted in advance at a ratio of [1:10] (1 ml eosin + 9 ml distilled water).

### Histomorphometric evaluation by light microscopy

In a blinded fashion, stained samples were photographed with the image processing program Axiovision® V 02-2002 Carl Zeiss (Carl Zeiss Microscopy Division, D-37081 Goettingen). For determination, the original local bone appeared light pink and homogeneous in the images, whereas the regenerated NB was more inhomogeneous and darker (Figs. 3A–D, 4A–C). NB areas facing toward the maxillary sinus lumen and subperiosteal NB toward the oral cavity were measured manually for each specimen by two experienced independent examiners that were trained before the evaluation.

### Fluorescence microscopy

Histological sections for FL were excited with Axio-phot® I (Carl Zeiss Microscopy Division, 37081 Göttingen, Germany) at wavelengths of 350 nm (blue), 594 nm (red) and 488 nm (green).

The maxillary sinus wall was divided into three zones (inner/up to the maxillary sinus lumen, middle/between, outer/up to the oral cavity). Each of these zones was assigned an extra value of 0–3 for each dye used, depending on its intensity (0 = no color recognizable, 1 = low luminosity, 2 = medium luminosity, 3 = high luminosity).

### Statistical analysis

The statistical evaluation of the histomorphometrically collected data was performed by using SigmaPlot 11.0 (Systat, Erkrath, Germany) and GraphPad Prism 5 (La Jolla, CA, USA). All data are reported as the means ± standard deviations.

Two-tailed Student’s t test was used in the parametric case. In the multisampling case, ANOVA with Sidak post hoc procedure was used. The level of significance was set at p ≤ 0.05 for all procedures. The significance level was corrected with the Bonferroni method to counteract the multiple test problem.

## Results

### Animal experiment

Reporting is performed according to the ARIVE guidelines. One animal was lost prior to surgery due to ventilation complications. Surgery and postoperative care were uneventful, and no side effects, such as wound healing disorders or local infections, could be observed.

### Histomorphometric analysis by light microscopy

Comparing the extent of the periosteal reaction (distance of the elevated periosteum, detected by subperiosteally formed bone, depicted in µm) measured on the inner layer of the maxillary sinus, no significant difference between the groups could be detected when considering both the surgical technique (p = 0.49) and the survival time (p = 0.55).

In terms of the periosteal reaction as the area of subperiosteally NB formation, no significant difference could be observed between the operation techniques (p = 0.31) or the survival time points (p = 0.28). In the BLC group, the periosteal reaction was demonstrably more pronounced than in the OSFE group, as demonstrated in Fig. 1. The 56-day samples also showed more NB formation than the 28-day samples for both techniques (Fig. 1).

To compare the two bone growth values reliably (evaluation of two sections per each maxillary sinus) per technique used (left maxillary sinus vs. right maxillary sinus) per animal, the values were sorted by maximum and minimum and compared with the surgical method. The smaller bone growth values showed a significant result with regard to the area. Here, BLC was superior to OSFE (p = 0.035). The other comparisons did not yield any significant differences.

### Histomorphometric analysis by fluorescence microscopy

**Fig. 5:**
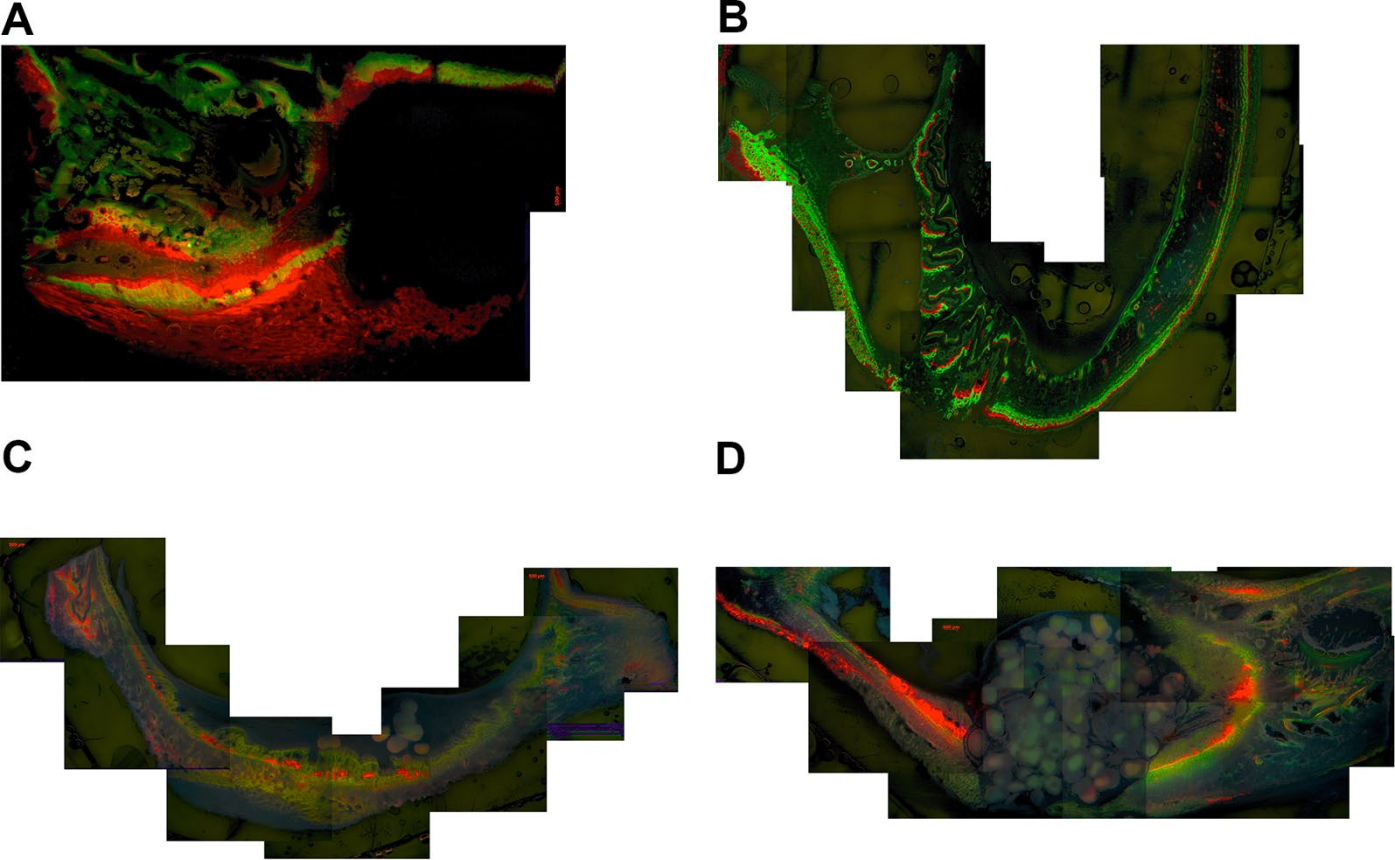
FL images according to the specimens of Fig. 2. **A** Sample of the BLC group at 28 days. The highest NB formation was measured after 8 days, as seen by the intense red fluorescence. Scale bar: 500 µm. **B** Sample of the OSFE group at 28 days. Fast initial bone growth, as seen by the intense green and red fluorescence. Scale bar: 500 µm. **C** Sample of the BLC group at 56 days. The highest activity was measured for Xylenol and Calcein blue, i.e., after 23 and 37 days. Scale bar: 500 µm. **D** Sample of the OSFE group at 56 days. The highest growth rates could be detected after 23 days. Scale bar: 500 µm

To determine the different bone growth behaviors, various fluorescent dyes at different time points were administered and analyzed in three regions of interest (inside, middle, outside—for details, see Fig. 5A–D).

The Friedman test showed a significant difference when the outside zone was examined. (p < 0.0001) R (median = 0) and Cg (median = 1) and between R and X (median = 1.5), with a range of 0.008 < p > 0.001).

Comparing the middle zone, significant differences were also detected (Friedman test p < 0.0001). Post hoc analysis revealed a significant difference in dye intensity between Cb (median = 0) and Cg (median = 2), between R (median = 0.5) and Cg and between Cb and X (median = 1), with p values of 0.000/0.003 and 0.000, respectively.

Again, a significant difference (p < 0.0001) could also be calculated by the Friedman test when the inner zone of the SF was compared. Post hoc analysis showed a significant group difference between Cb (median = 0.5) and Cg (median = 2), between R (median = 0) and Cg, between Cb and X (median = 2) and between R and X, with p values of 0.002/0.000/0.001 and 0.000, respectively (Fig. 1).

To determine the time- or location-dependent RBGR, different levels of fluorescence intensity were compared [23].

In particular, early administered colors Cg and X (administered at day 2/2 + 8 for Cg or at day 8/16 + 23 for X) showed significantly different growth behaviors. The subsequent Mann‒Whitney U tests calculated a slightly significant difference in the group comparison of the center and inside zones (p = 0.03) and center and outside zones (p = 0.02). The post hoc tests of the late applied yellow dye (R) did not reveal any significant group differences.

Of note, the balloon-assisted elevation (BASF) technique showed significantly more NB formation than the invasive instrumental guided detachment of the Schneiderian membrane by the open SL technique. In addition, no local or temporal correlation between bone growth and a specific zone could be demonstrated by FL.

## Discussion

Thanks to modern minimally invasive surgical techniques and optimized implant systems, the lack of sufficient bone in the maxillary posterior region can be counteracted by bone augmentation so that initial sufficient implant stability can be ensured later [3]. The BLC technique was developed to avoid rupture of the Schneiderian membrane during surgery and thus to simplify the process of membrane detachment. The mucous membrane of the maxillary sinus is separated from the bony bottom of the maxillary sinus by a minimally invasive, atraumatic and controlled balloon filled with liquid [30]. Because of this gentle procedure, the strain on the patient is extremely low [25].

However, the necessary minimum layer thickness of the residual bone in the case of simultaneous implantation and augmentation remains a controversial issue. In addition, disagreement exists over the choice of surgical approach, the most suitable augmentation material and the necessary healing time [22].

The long-term success of alternative therapy options, which do not require bone augmentation for the same indication position (e.g., subperiosteal framework implants, tilted implants or implants that have been anchored in the pterygoid process for a long time), is questionable, and considerable surgical risks are involved. Because of possible complications, such as massive inflammation and substantial consequential damage during implant removal, their use is questionable. Additionally, perforations of the maxillary SF, bleeding from the major palatinal artery and the pterygoid plexus, and because of the unfavorable implant axis in the medial-posterior direction, these techniques are not recommended [8].

In this study, we focused on the two techniques of SF elevation that are the easiest to implement and the most common and most successful today: the classic OSFE according to Tatum and the BASF technique.

For the evaluation of bone growth in the upper jaw, an animal species should be selected with human-like chewing forces and bone vascularization [11]. The current results are almost completely transferable to humans. Great importance should be attached to reproducibility under the same conditions. Therefore, the mini-pig is described as the animal model of choice [31, 34].

A variety of data have been presented on normal and pathological bone healing in mini-pigs, describing the comparable physiology of the bone tissue and the anatomical conditions similar to those of humans [10, 13, 31].

In contrast to the general surgical technique described in the introduction, surgical access was chosen via the lateral maxillary sinus to leave all teeth undamaged. In addition, the augmentation area was not exposed to chewing forces or "misbehavior" (compliance), which makes it reliable to compare the results. For this reason, the NB formation within the augmentations may have behaved differently from the NB formation in humans, in which the BAM or the implants are inserted at a site exposed to the physiological load of chewing.

Although even the loosening of the Schneiderian membrane triggers the formation of NBs as a stimulus, the necessity for BAMs to be used for the BLC approach has not yet been clarified [3, 15].

We chose the synthetic bone regeneration material “easy-graft™” for this experimental work because of the strong demand for alloplastic BAMs in routine clinical work, its ability for subsequent modeling and its simultaneous dimensional stability after healing. Furthermore, ß-TCP, which is the underlying compositional part of easy-graft™, seems to be a suitable graft material because of its wide range of applications, low complication rates and satisfying long-term results [5, 9, 12]. Furthermore, it is clinically approved and investigated in multiple experimental and clinical studies.

We assumed that the inserted osteoconductive BAM merely acts as a placeholder or functions as a framework. The material introduced, therefore, plays a less significant role with regard to a periosteal reaction than the technique or the surgical procedure.

The aim of the present study was to quantify the periosteal response after the respective surgical method had been performed to compare the two SF elevation techniques. A realistic evaluation of the morphology of the cells and the differentiation of the various tissues was ensured by Giemsa eosin staining.

If one considers the periosteal reaction as an area, then a difference can be shown in the available healing time of the augmentations (Fig. 1). Although not significant, for the BLC technique, the periosteal reaction was demonstrably more pronounced than after OSFE. More NB formation could also be detected for the longer survival time point (T2).

This means that the longer healing time of the augmentations (56 days) with the applied technique of the OSFE in relation to the periosteal reaction did not allow the bone growth values that were achieved with the BLC technique by the same healing time (56 days). Even a comparison of the techniques with a shortened healing time (28 days) showed that the area of the periosteal reaction was larger in the BLC group than in the OSFE group. The BLC technique is thus superior to OSFE in terms of bone turnover. Of note, despite the low number of cases, the gradient indicates a definite trend (Fig. 1).

To show whether the surgical technique had a significant influence on the maximum or minimum expected bone growth area, values were compared by FL (Fig. 1). Especially in situations in which rapid initial healing of the augmentation or quick bone regeneration in the first days after the operation is desired, the BLC technique seems to provide better initial results in terms of NB than OSFE. Thus, the BLC technique induces faster bone growth.

The FL enabled the temporal course of bone metabolism to be objectified, and thus, the formation of NBs could be assigned to a highly specific formation time.

With regard to the course of time, bone growth behavior does not seem to be related to a specific zone, and no temporal connection could be determined. However, strikingly, the initial NB fractions were significantly superior to those formed later, as revealed by the two dyes that were administered initially. Nevertheless, the duration of the healing phase played a greater role when considering maximum bone growth than the surgical technique.

Following Cb staining, 13 out of 14 samples in the central region showed no bone growth at all. In the other two ROIs, 50% of the samples also showed no bone growth. One assumes that the middle region was inferior to the other two regions with regard to bone turnover at the applied time point (Fig. 5A–D).

This suggests that, at least from the 16th day or from the 30th day after surgery, no significant bone growth occurred in the investigated zone.

Notably, the present study did not show any particular growth behavior in relation to a specific zone. Each zone showed its own behavior, which seemed to be independent of each other in terms of location and time. If NB was formed, it created the impression that this process started at the site of the set stimulus and went into deeper regions. Moreover, the duration of healing had more influence on the maximum NB formation than the surgical technique.

With the BLC technique in combination with a long healing time, better NB formation could be achieved than with the OSFE. Various reasons for this can be proposed.

The maintenance of a sufficient vascular supply system during the elevation of the mucous membrane (mainly the stratum vasculare of the lamina propria) might play a decisive role in the success of SF elevation. Although the lifted mucous membrane has a sufficient vascular network for its supply [21], larger venous blood vessels that pass from the bone of the SF into the Schneiderian membrane are torn off during elevation. Studies have demonstrated that the mucous membrane when directly removed with instruments shows microflap formation with partial detachment or tearing of the periosteum [29]. Additionally, when the mucosa is removed by the seemingly gentle piezo-electric technique, the micro vessels are torn off by constant vibrations, microthrombi are induced, and thus, the blood supply is highly compromised or even interrupted [14, 30]. The mucosa, when lifted off with the balloon, on the other hand, remains absolutely intact and unstressed. This effect can also be seen in the histological images depicted in Figs. 3 and 4.

The BLC technique is, therefore, a promising procedure that facilitates and standardizes surgery even under difficult anatomical conditions (septa) and inaccessible access paths. Furthermore, it supports bone turnover by its gentle method and, therefore, provides demonstrably higher NB formation values compared with the open instrumental technique.

The decision for the "right" access route (lateral sinus wall or transcrestal access) depends on the individual clinical situation and, in particular, on the quantity and quality of the preexisting crestal bone site. The use of BAMs for lateral access is a well-documented procedure with a low complication rate of only approx. 5%. The BLC approach, however, is also a component of many examinations and represents an alternative to external procedures because of its minimal invasiveness [35].

The present study demonstrates that the surgical procedure can contribute to favorable conditions for the formation of NB and moreover for the RBGR or for the healing of augmentations. Although several studies have suggested that the search for the perfect BAM is the sole item that requires adjustment in the system of SF elevations, this is to be questioned and reconsidered because various studies have revealed sufficient bone turnover in the maxillary sinus even without BAMs [35]. In addition to the treatment factors, systemic factors and the condition of the patient's bone position must also be taken into account as factors influencing NB formation.

When comparing clinical studies in which SF augmentations with alloplastic BAMs have been performed, Zerbo et al. [36] and Frenken et al. [7] have shown NB formation rates of approximately 17% after an integration phase of 6–8 months for augmentation with ß-TCP-based BAM and approximately 27% for augmentation with a biphasic calcium-phosphate based BAM.

In summary, the highest bone formation values were seen in the first weeks shortly after augmentation, especially for the BLC technique. However, the bone formation values approached each other over time. In the 56-day group, the treatment group showed significantly faster initial growth behavior than the OSFE group (p < 0.02).

Almost twice as much bone was initially newly formed with the BASF technique, suggesting that a sufficient blood supply is a prerequisite for further bone regeneration. The detachment of the SF mucosa by the BLC technique, which is gentle on the blood vessels, thus has a clearly positive effect on the initial phase of osseointegration of the BAM.

Most authors agree that, for sufficient stable osseointegration of dental implants in the area of the augmented maxillary sinus, NB formation of 20–35% should be achieved at minimum [1, 19, 24, 26].

Both the BLC and the OSFE provide good results in terms of the amount of NB formation based on an osteoconductive BAM. The surgeon's task is therefore to assess the individual clinical situation and then select the appropriate surgical method to achieve optimal results.

## Conclusion

In the investigation of the preparations concerning a possible influence of the surgical technique on the extent of NB formation and the healing process of the augmentations, in direct comparison with the open sinus lift technique there was a trend of certain superiority of the BLC technique as demonstrated:

- The BLC technique induces a lower selective pressure on the Schneiderian membrane, so the vascular stress of the latter is reduced compared to that of OSFE.
- Because of the homogeneous distribution of pressure on the mucosa, the BLC technique might give better NB formation values in total than OSFE
- In this animal study the BLC technique gave better initial results in terms of NB formation than OSFE.

However, with regard to the possible influence of the surgical technique on the long-term amount of NB formation, both techniques proved to be of equal value. Conversely, the BLC procedure can be expected to have a lower surgical complication rate.

## Data Availability

The acquired data sets created and analyzed during the current study are available from the corresponding author upon request.

## Abbreviations

SF: Maxillary sinus floor
BAM: Bone augmentation material
BASF: Balloon-assisted SF elevation
OSFE: Open lateral sinus wall approach (control group, CG)
BLC: Balloon-lift-control system (treatment group, TG)
NB: New bone formation
RGBR: Relative bone growth rate
SL: Sinus lift
TG: Treatment-group
CG: Control group
PLGA: Copolymer poly(lactic-co-glycolic acid)
β-TCP: Synthetic bone augmentation material β-tricalcium phosphate
ANOVA: Analysis of variance, statistical method
MMA: Methyl methacrylate, embedding material
FL: Fluorescent labeling
Cg: Calcein green, fluorochrome
X: Xylenol orange, fluorochrome
Cb: Calcein blue, fluorochrome
R: Rolitracycline, fluorochrome
